# Photoperiod affects the laying performance of the mountain duck by regulating endocrine hormones and gene expression

**DOI:** 10.1002/vms3.508

**Published:** 2021-05-06

**Authors:** Hongjia Ouyang, Bo Yang, Yongcong Lao, Jun Tang, Yunbo Tian, Yunmao Huang

**Affiliations:** ^1^ Guangdong Province Key Laboratory of Waterfowl Healthy Breeding College of Animal Science and Technology Zhongkai University of Agriculture and Engineering Guangzhou China

**Keywords:** GnIH, laying rate, melatonin, photoperiod, reproduction

## Abstract

Light mainly affects animal reproductive performance through the hypothalamus‐ pituitary‐gonadal axis, but the specific regulating mechanism is not yet clear in duck. To reveal the effects of light on the laying performance of ducks and its possible regulatory mechanisms, Shanma ducks at 52 weeks age were divided into three group treated with different photoperiods of 16 hr (control group), 24 hr (long‐photoperiod group, LP), and 8 hr (short‐photoperiod group, SP). Laying performance, endocrine‐related hormones and gene expression of three groups were compared. The results showed that laying performance was greatest in the LP group; including laying rate, average egg weight and feed‐egg ratio. Compared to the SP group, GnIH plasma concentration was decreased in the LP group, whilst FSH was increased in the LP group. GnIHR gene expression in the pituitary and large yellow follicles were downregulated in the LP group. The expression of Mel‐a in large white follicles, and Mel‐b and Mel‐c in the hypothalamus were also downregulated in the LP group. Altogether these results suggest that extended photoperiods may promote the laying performance of ducks by inhibiting the secretion of GnIH and the expression of GnIHR and melatonin receptor genes.

## Graphical abstract

This study found that the average laying rate and the average egg weight of ducks in the LP group both increased. Extending photoperiods may improve the laying performance of ducks by reducing plasma levels of GnIH hormone and the expression of GnIHR and melatonin receptor genes.

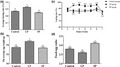

## 1 INTRODUCTION

Reproductive activity in animals is regulated by the neuroendocrine system dominated through the hypothalamus‐pituitary‐gonadal axis (HPGA) (Greives et al., 2007; Schlatt et al., 2016). The HPGA forms a closed self‐feedback system under the regulation of the nervous system, and the hypothalamus, pituitary and gonads cooperate or restrict each other to precisely regulate the development and function of the reproductive system (Campbell et al., 1999; Kaprara & Huhtaniemi, 2017). Light is a key environmental factor in the regulation of reproductive activity in animals, mainly through its effects on the HPGA (Lewis, 2006). Increasing photoperiods stimulate the secretion of gonadotropin‐releasing hormone (GnRH) in the hypothalamus, which promotes the secretion of luteinising hormone (LH) and follicle‐stimulating hormone (FSH), and finally induces gonadal maturation and reproductive behaviour (Bedecarrats et al., 2016).

Melatonin is an important hormone involved in the detection of changes in light; its secretion is affected by photoperiod, showing diurnal and seasonal secretion, and it mediates the regulation of reproductive performance in animals (Renuka & Joshi, 2010; Ubuka et al., 2013). Gonadotropin‐inhibitory hormone (GnIH) is a negative regulator of reproductive activities, which is effective at multiple levels in the HPAG, thus maintaining the stability of the reproductive endocrine system and coordinating animal growth and reproduction (Bedecarrats et al., 2016).

Previous studies have found that melatonin may play a role in regulating animal reproduction by promoting GnIH, but the specific mechanism is still unclear (Ubuka et al., 2005). The illumination time of Shanma duck in the laying period is usually 16 hr. In this study, we used 16 hr of light as a control group to increase or decrease the laying time of laying ducks and then compared their egg laying performance, hormone levels and expression levels of endocrine‐related genes. We focus on the changes in melatonin and GnIH, to explore their relationship and role in the photoperiod regulation of reproduction.

## 2 MATERIALS AND METHODS

### 2.1 Animals and experimental design

A total of 120 female Shanma ducks (52 weeks of age) were obtained from the Duck Breeding Farm. The animal trials took place at the Animal Experiment and Breeding Center of Zhongkai University of Agriculture and Engineering.

All ducks were housed together with exposure to a photoperiod of 16 hr for 1 week; they were subsequently divided into three equal groups (*n* = 40). The control group, long‐photoperiod (LP) group and short‐photoperiod (SP) group were exposed to photoperiods of 16, 24 and 8 hr, respectively. The experiment lasted a total of 54 days. During the experiment, egg production and feed consume for each group were recorded. Meanwhile, the temperature, humidity and light intensity in the duck house were monitored and recorded. Diet formula and nutrient components are listed in the supporting information Tables S1 and S2.

### 2.2 Detection of ovarian follicle development and tissues collection

At d1 and d54, 16 ducks were randomly selected from each group for live weighing. Amongst them, eight ducks of each group were used for the collection of serums, tissues and examination of ovarian follicle development. All ducks were euthanised by decapitation for tissues collection. The hypothalamus, pituitary and ovarian tissues were collected, immediately frozen in liquid nitrogen and stored at −80°C prior to use. Weigh the ovarian tissues, and large white follicles (LWF; white follicles greater than 5 mm in diameter), small yellow follicles (SYF; yellow follicles less than with 10 mm in diameter), and large yellow follicles (LYF; yellow follicles greater than 10 mm in diameter) were counted and collected.

### 2.3 Hormone assay

Plasma concentrations of GnRH, GnIH, MT, FSH, LH, PRL, E2, P4, T3 and T4 were determined using their corresponding ELISA kits (SUER Co. Ltd). For all ELISA assays, the intra‐assay and interassay coefficients of variation were set at <15%, and the *r*‐values of the standard curves were >0.99.

### 2.4 Total RNA isolation, cDNA synthesis and quantitative real‐time PCR (qRT‐PCR)

Total RNAs were isolated using Trizol Reagent (Invitrogen), following the manufacturer's instructions. The quality and quantity of RNA samples were detected by 1.5% agarose gel electrophoresis and absorbance OD (optical density) at 260/280 nm ratio, respectively. The cDNA synthesis was performed using a RevertAid First Strand cDNA Synthesis Kit (Fermentas) with random hexamers.

The qRT‐PCR was performed using SYBR Green PCR Master Mix (Invitrogen) in a total reaction volume of 20 μl, in an ABI7500 system (ABI) under the following conditions: 95°C for 3 min, 40 cycles of 95°C for 10 s, annealing temperature (56–62°C) for 30 s and 72°C for 30 s and a final extension at 72°C for 1 min. All reactions were run in triplicate and presented as means ± standard error means (*SEM*). The relative expression levels of the gene were calculated using the comparative 2^−ΔΔCT^(CT is threshold cycle) method as previously described (Livak and Schmittgen, 2001).

Primers used for qRT‐PCR were designed using Premier Primer 5.0 software (Premier Bio‐soft International) and synthesised by BIG Co. Ltd. The *β‐actin* gene was used as the reference gene. Details of primers are summarised in Table 1.

**TABLE 1:** Detailed information of primers

Gene name	Nucleotide sequences (5′→3′)	Annealing temperature (℃)	Accession number
*GnRH*	F: GGCTCAGCACTGGTCTTAT	60	XM_013104148.2
	R: TCTGCACTTCCCCTATCTT		
*GnRHR*	F: TCTGCTGGACCCCCTACTAC	60	XM_021268169.1
	R:TCCAGGCAGGCGTTGAAGAG		
*GnIH*	F: TTTATCACAGAGGCTTGGG	58	XM_005024271.3
	R: GTTCAGATTCCTGGACACC		
*GnIHR*	F: GTTGTCATGTACACCCGCAT	62	XM_005028365.3
	R: TCCTGCGAGACACCTTCCTC		
*PRL*	F: CTGACAAAGGAAGGAGTGA	58	NM_001310372.1
	R: TTCTGAAGAGAGGAAATGG		
*VIP*	F: TCAAACGCCACTCTGATGCT	58	XM_005024791.3
	R: GAGGGGTTTAGCTCTTCCTGG		
*Mel‐1a*	F: ATTAGTGGCTTCTTGATGGGC	62	XM_021279436.1
	R: CAAACAGGTTGGGCACGATA		
*Mel‐1b*	F: TCGATGGCTACTGCTAGTCCT	60	XM_021267727.1
	R: CCGAGACTGAAGCCAAGTGA		
*Mel‐1c*	F: ACCGCCATCGCAATCAAC	62	XM_013100494.2
	R: GCAAGGACCCAACGAAGAA		
*β‐actin*	F: ATGTCGCCCTGGATTTCG	56–62	NM_001310421.1
	R: CACAGGACTCCATACCCAAGAA		

Note

F represents the forward primer; R represents the reverse primer.

### 2.5 Statistical analyses

Statistical analyses were performed using IBM SPSS software (ver. 19.0; IBM SPSS). Values are presented as means ± *SEM*. The threshold for significance was set at *p* < 0.05 and for high significance at *p* < 0.01.

## 3 RESULTS

### 3.1 Body weight

**FIGURE 1:**
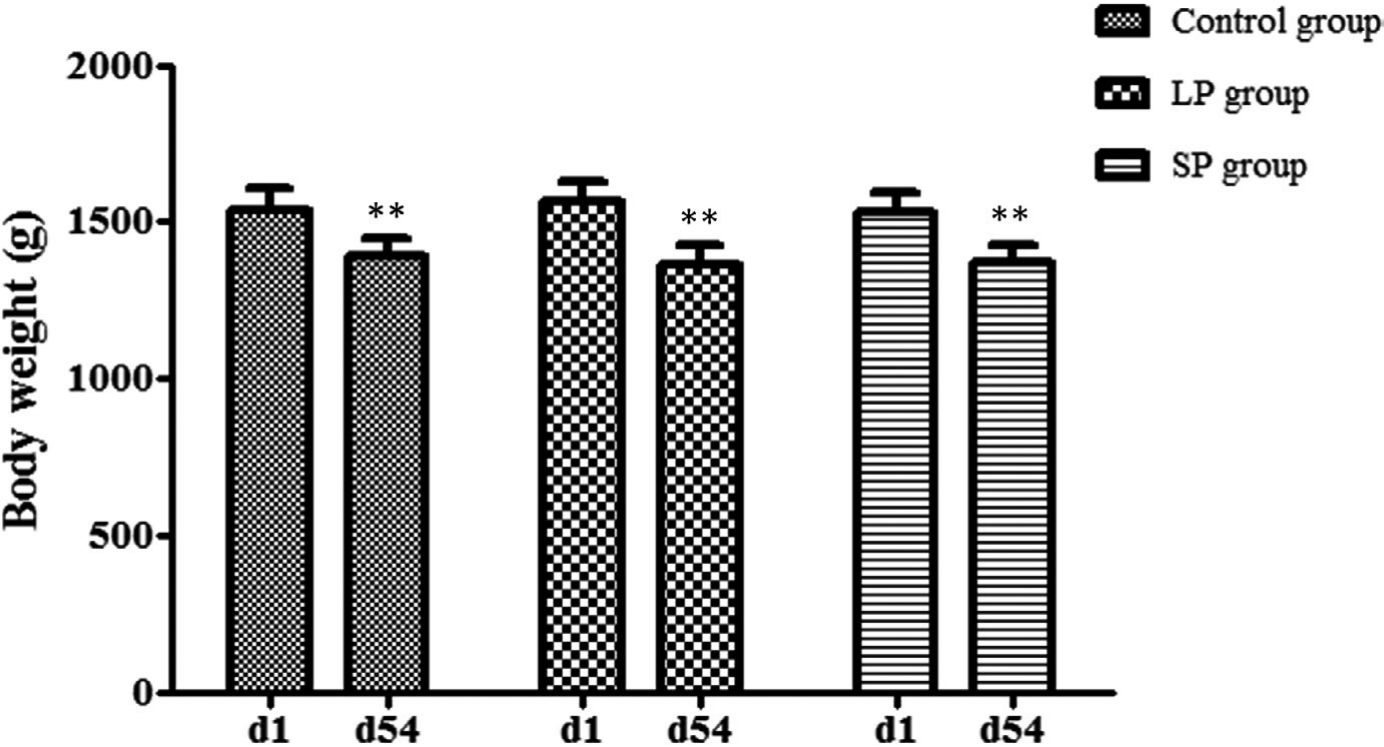
The average body weight of the LP, SP and control groups. *p < 0.05; **p < 0.01

After different durations of light treatment (at 54 days), the average body weights amongst groups LP, SP and control were no significant difference (*p* > 0.05). However, the body weight of each group was lower than (*p* < 0.01) that at 1 day (Figure 1). Body weight of the LP group decreased by 12.65%, more than the SP group (10.52%) and control group (9.64%).

### 3.2 Laying performance

**FIGURE 2:**
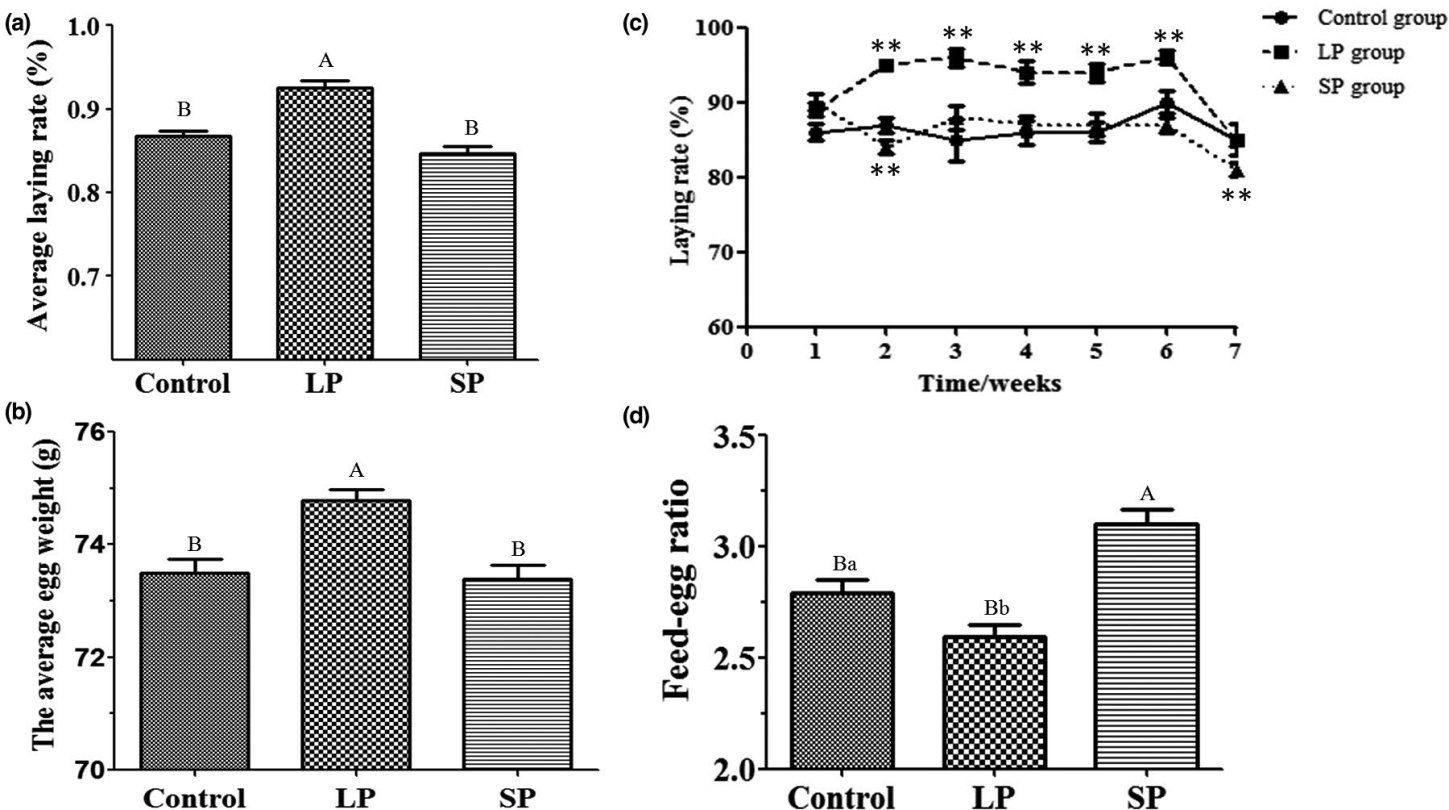
The laying performances of the LP, SP, and control groups. (a) Average laying rate. (b) Average egg weight. (c) The laying rate of each week during the trial period. (d) Feed‐egg ratio. *p < 0.05; **p < 0.01; ^a,b^ *p* < 0.05; ^A,B^ *p* < 0.01

During the trial period, the average laying rate and the average egg weight of the LP group were both higher than (*p* < 0.01) that of the SP and control groups (Figure 2a and b). Compared with ducks before dimming (at d 1), the egg production rate of the LP group increased significantly (*p* < 0.05) during the 2–6 weeks (Figure 2c). Egg production rate in the SP group decreased significantly in the second week (*p* < 0.05), and then began to return during the third week (Figure 2c). In the later period of the experiment (d 43–d 54), egg production rate of all three groups decreased rapidly, with the SP group showing the greatest decline (*p* < 0.01; Figure 2c).

Calculations and comparisons of the feed conversion ratios of the three groups found that the feed‐egg ratio of the LP group was lower than that of the SP control groups, and in addition, the results suggested that long light periods may be conducive to promoting the feed conversion rate of laying ducks (Figure 2d). Comparing the ovarian follicular development of the three groups, there was no significant difference in the number of large yellow follicles, small yellow follicles and large white follicles amongst the three groups, which indicated that light had no obvious effect on the ovarian follicles of mature egg ducks (Table 2).

**TABLE 2:** The development of the ovaries in the LP, SP and control groups

Groups	Control	LP	SP
Gonadal index	4.25 ± 0.17	4.21 ± 0.16	4.33 ± 0.15
Numbers of LYF	4.60 ± 0.27	4.88 ± 0.23	4.30 ± 0.29
Numbers of SYF	4.60 ± 0.87	4.55 ± 0.78	5.86 ± 0.51
Numbers of LWF	7.44 ± 4.10	7.89 ± 0.86	10.14 ± 0.80

Note

Gonadal index = ovarian tissues weigh/body weight.

### 3.3 Plasma hormone levels

**FIGURE 3:**
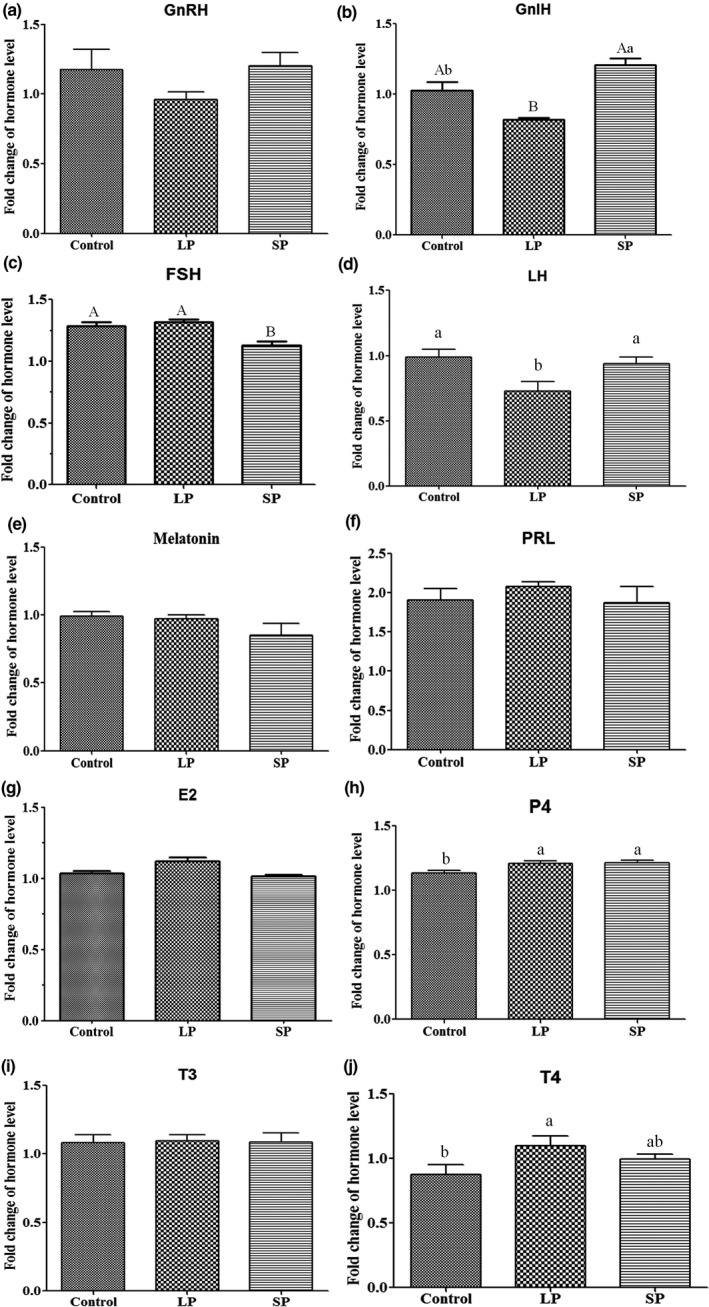
The fold change of plasma hormone level of the LP, SP, and control groups between d 1 and d 54. (a) GnRH; (b) GnIH; (c) FSH; (d) LH; (e) Melatonin; (f) PRL; (g) E2; (h) P4; (i)T3; (j) T4. ^a,b^ *p* < 0.05; ^A,B^ *p* < 0.01

**FIGURE 4:**
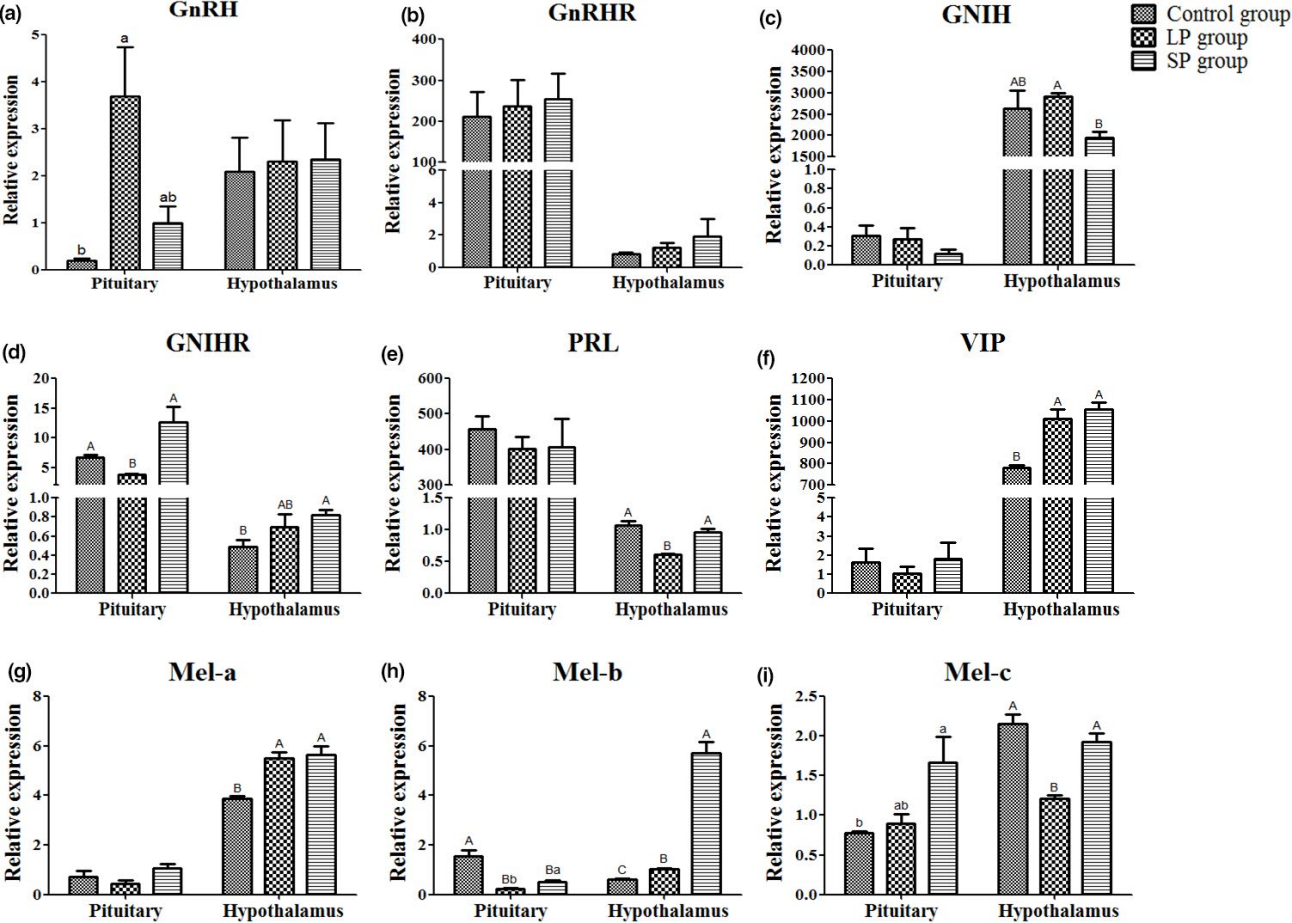
The mRNA level of reproductive endocrine‐related genes in the pituitary and hypothalamus. (a) GnRH; (b) GnRHR; (c) GnIH; (d) GnIHR; (e) PRL; (f) VIP; (g) Mel‐a; (h) Mel‐b; (i) Mel‐c. ^a,b^ *p* < 0.05; ^A,B^ *p* < 0.01

Plasma concentrations of GnRH, GnIH, MT, FSH, LH, PRL, E2, P4, T3 and T4 were determined at experimental d 1 and d 54, and their fold changes are shown in Figure 4. Compared to the control group, the fold change in GnIH was decreased in the LP group (*p* < 0.01) and increased in the SP group (*p* < 0.05), whilst the fold change of GnRH showed no significant difference amongst the three groups (Figure 3a and b). Plasma concentrations of FSH were upregulated at d 54 in all three groups, with a lower fold change in the SP group (*p* < 0.01) than that in the control and LP groups (Figure 3c). The fold change of LH was lower in the LP group (*p* < 0.01) than that in the control and SP groups (Figure 3d). Plasma concentrations of MT were downregulated at d 54 in the SP group, but there was no significant difference amongst the three groups (Figure 3e). Fold changes in PRL and E2 amongst the three groups were also not significant (Figure 3f and g). Plasma concentrations of P4 were upregulated at d 54 in all three groups, with fold changes larger in the LP and SP groups (*p* < 0.05) than in the control group (Figure 3h). For T3and T4, the fold change between the three groups was not significant in T3; however, the fold change inT4 was greater in the LP group (*p* < 0.05) than that in the control group (Figure 3i and j).

### 3.4 Gene expression level

After different durations of light treatment (at 54 d), the gene expression of reproductive endocrine genes was detected in the hypothalamus, pituitary and ovarian tissues. Compared to the control group, the expression of GnRH in the pituitary was increased in the LP group (*p* < 0.05), and the expression of its receptor GnRHR showed no significant difference amongst the three groups in the pituitary or hypothalamus (Figure 4a and b). The mRNA level of GnIH in the hypothalamus was higher (*p* < 0.01) in the LP group than in the SP group (Figure 4c), whilst the expression of GnIHR in the hypothalamus was higher (*p* < 0.01) in the SP group and lower (*p* < 0.01) in the LP group compared to the control and SP groups (Figure 4d). Compared to the control group, the expression of PRL in the hypothalamus was decreased (*p* < 0.01) in the LP group, and the expression of VIP was increased (*p* < 0.01) in both the LP and SP groups (Figure 4e and f). For the three different subtypes of melatonin receptors Mel‐a, Mel‐b and Mel‐c, the results showed that the mRNA levels of Mel‐b and Mel‐c in the hypothalamus were higher (*p* < 0.01) in the SP group than in the LP group. In the pituitary, the expression of Mel‐b was highest (*p* < 0.01) in the control group and also was higher (*p* < 0.05) in the SP group than in the LP group (Figure 4g–i).

**FIGURE 5:**
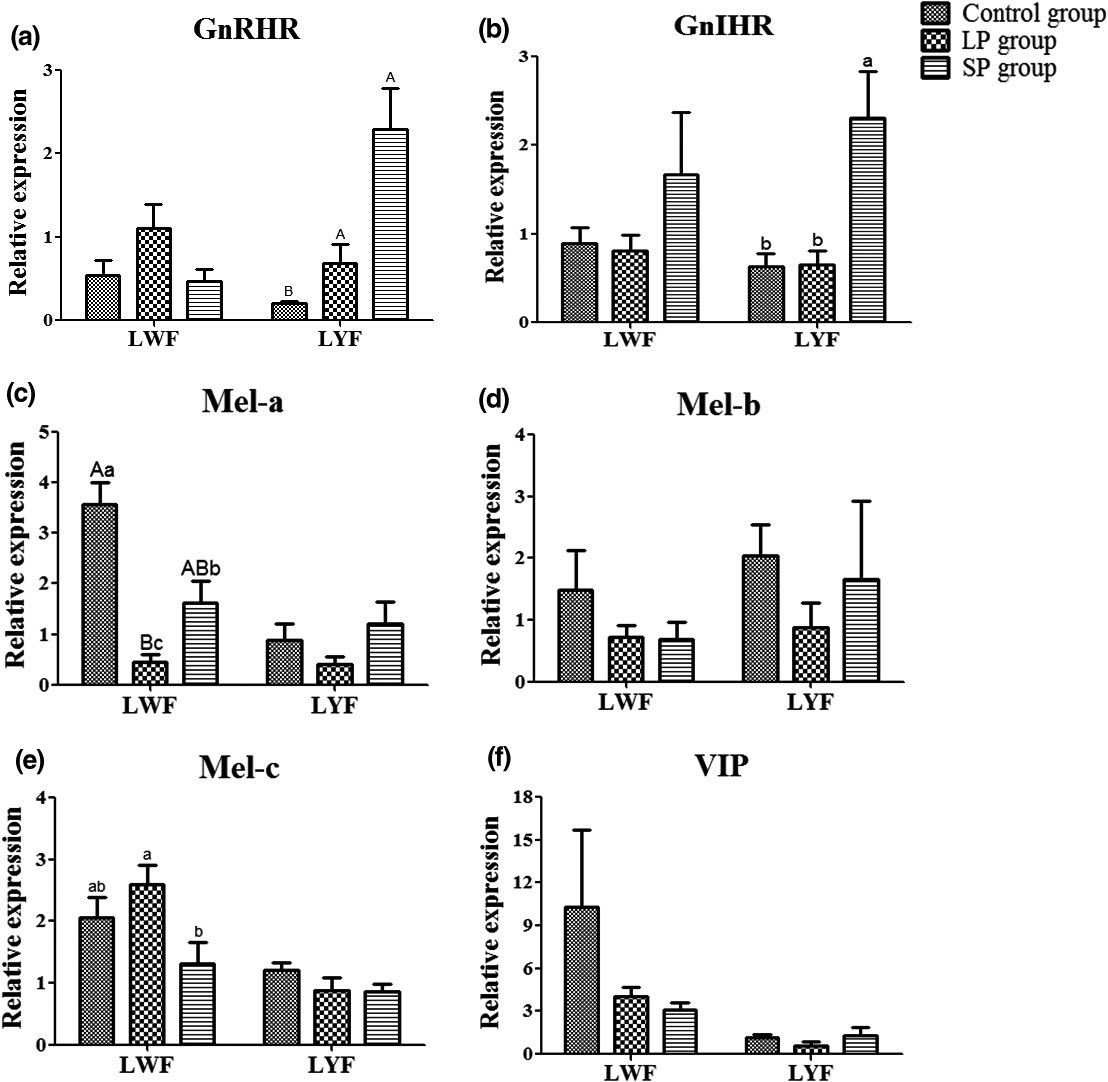
The mRNA level of reproductive endocrine‐related genes in large yellow follicles and large white follicles. (a) GnRH; (b) GnRHR; (c) GnIH; (d) GnIHR; (e) PRL; (f)VIP; (g) Mel‐a; (h) Mel‐b; (i) Mel‐c. ^a,b^ *p* < 0.05; ^A,B^ *p* < 0.01

In ovarian tissues, the mRNA level of these genes was detected in LWF and LYF, respectively. The results showed that GnRH, GnIH and PRL were not expressed or hardly expressed in the LWF and LYF of all three groups. The expression of GnRHR in LYF was increased (*p* < 0.01) in the LP and SP groups compared to the control group, and the mRNA level of GnIHR in LYF was higher (*p* < 0.05) in the SP group than in the control and LP groups (Figure 5a and b). In LYF, the mRNA levels of melatonin receptors Mel‐a, Mel‐b and Mel‐c were not significantly different amongst the three groups (Figure 5c–e). The difference in VIP mRNA level between the three groups was not significant (Figure 5f). However, compared to the control group, the expression of Mel‐a was decreased in the LP (*p* < 0.01) and SP (*p* < 0.05) groups and was higher (*p* < 0.05) in the SP group than in the LP group (Figure 5c).

## 4 DISCUSSION

Light is an important environmental factor, which affects physical maturation, egg production and fertilisation of poultry (Nakane & Yoshimura, 2018). We found that a long photoperiod could improve the laying performance of ducks; the average laying rate and the average egg weight of the LP group during the experiment were significantly higher than those in the control and SP groups. In white Roman geese, it has also been reported that long photoperiods can increase the average weight of eggs (Wang et al., 2009). Interestingly, the current results showed that the feed‐egg ratio of the LP group was significantly lower than those of the control and SP groups. Throughout the whole experiment, the laying performance of the LP group including laying rate, egg weight and feed‐egg ratio was superior to the SP and control groups. However, weight loss for the LP group was greatest as egg production demands the mobilisation and consumption of body energy. Furthermore, prolonged photoperiods also increase the activity of laying ducks, which may have resulted in greater weight loss in the LP group (Gous et al., 2000).

Photoreceptors exist in the hypothalamus of poultry, which convert external light signals into nerve impulses and act on the HPGA, regulating related hormone secretion and gene expression thereby affecting reproductive activity (Dawson et al., 2000). When comparing hormone levels amongst the LP, SP and control groups, we found that the plasma concentrations of GnIH were decreased in the LP group and increased in the SP group. Plasma concentrations of FSH were upregulated at d 54 in all three groups, with the greatest increase in the LP group. Other important reproductive hormones include GnRH, E2 and P4, and for these there was no significant difference between the LP and SP groups. Therefore, we suggest that the improvement of egg production performance in the LP group was probably due to a decrease in GnIH hormone. GnIH plays a negative regulatory role in animal reproductive activities (Tsutsui et al., 2013), whilst FSH promotes reproductive activities and promotes oestrogen synthesis and ovarian follicular growth (De Pascali et al., 2018). We suggest that the decrease in GnIH hormone promoted FSH secretion and eventually improved egg production performance in this study.

Photoperiods regulate the expression of the GnIH gene via a melatonin‐dependent process (Tsutsui et al., 2000; Ubuka et al., 2005). The latter authors detected the expression of reproductive endocrine‐related genes in HPGA tissue and determined that long photoperiods promote expression of the GnIH gene in the hypothalamus; this differs from the results of GnIH plasma hormone levels. However, the current study found that the expression level of the GnIHR gene (GPR147) in the pituitary and LYF was lower in the LP group than in the SP group. The GnIH gene works by binding to its specific receptor (GPR147) (Ubuka et al., 2008; Yin et al., 2005). Thus, low expression of the GnIHR gene in the SP group may be the cause of the improved laying performance.

Melatonin is also regulated by photoperiod (Zhang et al., 2017), which increases the expression of GnIH in quail (Chowdhury et al., 2010; Ubuka et al., 2005), and inhibits reproductive activities in quail and chicken (Guyomarc'H et al., 2001; Rozenboim et al., 2002). There was no significant difference in mRNA and plasma hormone levels of melatonin amongst the three groups of ducks in this experiment. However, after detecting the three melatonin receptor genes, we found that the gene expression levels of Mel‐b and c in the hypothalamus of the SP group significantly increased, and the expression level of Mel‐a in the white follicles was significantly upregulated. It is possible that more melatonin bound to receptors in the SP group and inhibited laying performance.

## 5 CONCLUSIONS

In summary, we found that the average laying rate and the average egg weight of ducks in the LP group both increased. Therefore, extending photoperiods may improve the laying performance of ducks by reducing plasma levels of GnIH hormone and the expression of GnIHR and melatonin receptor genes.

## CONFLICT OF INTEREST

This manuscript has no conflicts of interest.

## AUTHOR CONTRIBUTIONS

HJOY: Conceptualization, Investigation, Formal Analysis and Writing‐Original Draft Preparation; BY: Investigation, Formal Analysis and Writing‐Original Draft Preparation; YCL: Investigation and Formal Analysis; JT: Resources and Formal Analysis; YBT: Conceptualization and Writing‐Review and Editing; YMH: Conceptualization, Writing‐Review and Editing and Funding Acquisition.

## ETHICS STATEMENT

This experiment was performed in accordance with the regulations and guidelines of the Animal Care Committee of the Zhongkai University of Agriculture and Engineering in China, and all efforts were made to minimise animal suffering.

### PEER REVIEW

The peer review history for this article is available at https://publons.com/publon/10.1002/vms3.508.

## Supporting information

Table S1‐S2
