# Supplementary material for: Photoperiod affects the laying performance of the mountain duck by regulating endocrine hormones and gene expression
Source: Vet Med Sci. 2021 May 6;7(5):1899–906. doi: 10.1002/vms3.508 (PMC8464274; doi:10.1002/vms3.508)
Supplement: Supplementary file 1 — Table S1‐S2 [file VMS3-7-1899-s001.doc]

Table S1. Feed formula for ducks

| Formula | % by weight |
| --- | --- |
| Corn | 49.5% |
| Soybean meal | 18% |
| Fish meal | 3% |
| Rapeseed cake | 6% |
| Wheat bran | 14% |
| Calcium hydrophosphate | 1.2% |
| Conch meal | 7% |
| Salt | 0.3% |
| Additive | 1% |

Table S2. Mainly nutritional components of diet.

| Components | % by weight |
| --- | --- |
| Crude protein | 18.29 % |
| Metabolizable energy | 11.38 MJ/kg |
| methionine | 0.39% |
| lysine | 0.91% |
| calcium | 2.97% |
| Available phosphorus | 0.39% |
